# Minimizing adverse effects of Cerenkov radiation induced photodynamic therapy with transformable photosensitizer-loaded nanovesicles

**DOI:** 10.1186/s12951-022-01401-0

**Published:** 2022-04-27

**Authors:** Ruijie Qian, Kun Wang, Yawen Guo, Hongyan Li, Ziyang Zhu, Xiaojuan Huang, Chengpeng Gong, Yu Gao, Rong Guo, Biao Yang, Chenyang Wang, Dawei Jiang, Xiaoli Lan, Rui An, Zairong Gao

**Affiliations:** 1 Department of Nuclear Medicine, Union Hospital, Tongji Medical College, Huazhong University of Science and Technology, No. 1277 Jiefang Ave, Wuhan, 430022 China; 2 Hubei Key Laboratory of Molecular Imaging, Wuhan, 430022 China; 3 Department of Oncology, Tongji Medical College, Tongji Hospital, Huazhong University of Science and Technology, Wuhan, China; 4 Department of Nuclear Medicine, Sir Run Run Shaw Hospital, Zhejiang University School of Medicine, Hangzhou, 310000 China

**Keywords:** Cerenkov radiation, Exosome coating, Nuclear medicine, Nanomedicine, Photodynamic therapy, Synergistic therapy, Breast cancer

## Abstract

**Background:**

Photodynamic therapy (PDT) is a promising antitumor strategy with fewer adverse effects and higher selectivity than conventional therapies. Recently, a series of reports have suggested that PDT induced by Cerenkov radiation (CR) (CR-PDT) has deeper tissue penetration than traditional PDT; however, the strategy of coupling radionuclides with photosensitizers may cause severe side effects.

**Methods:**

We designed tumor-targeting nanoparticles (^131^I-EM@ALA) by loading 5-aminolevulinic acid (ALA) into an ^131^I-labeled exosome mimetic (EM) to achieve combined antitumor therapy. In addition to playing a radiotherapeutic role, ^131^I served as an internal light source for the Cerenkov radiation (CR).

**Results:**

The drug-loaded nanoparticles effectively targeted tumors as confirmed by confocal imaging, flow cytometry, and small animal fluorescence imaging. In vitro and in vivo experiments demonstrated that ^131^I-EM@ALA produced a promising antitumor effect through the synergy of radiotherapy and CR-PDT. The nanoparticles killed tumor cells by inducing DNA damage and activating the lysosome-mitochondrial pathways. No obvious abnormalities in the hematology analyses, blood biochemistry, or histological examinations were observed during the treatment.

**Conclusions:**

We successfully engineered a nanocarrier coloaded with the radionuclide ^131^I and a photosensitizer precursor for combined radiotherapy and PDT for the treatment of breast cancer.

**Graphical Abstract:**

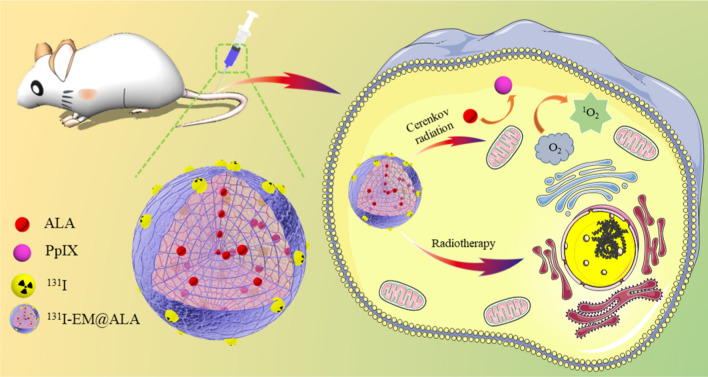

**Supplementary Information:**

The online version contains supplementary material available at 10.1186/s12951-022-01401-0.

## Background

Photodynamic therapy (PDT) is already a clinically approved and promising therapeutic modality for the treatment of neoplastic and non-malignant disease [1]. PDT involves irradiating photosensitizers (PSs) with light of specific wavelengths to trigger the generation of reactive oxygen species (ROS), which in turn cause cancer cell death [2]. However, PDT has been confined to superficial tissues owing to the rapid attenuation of light in tissue and the concomitant limited tissue penetration [3]. Several strategies have been designed to address this issue, including those based on near-infrared light, self-luminescence, X-ray radiation, and Cerenkov radiation (CR) [4–7]. In Cerenkov radiation-induced PDT (CR-PDT), photosensitizers are activated by CR produced by nearby radionuclides to generate damaging ROS, making it a promising type of PDT for addressing the depth dependency [5, 8, 9]. However, this phenomenon, also known as Cerenkov resonance energy transfer (CRET) [3, 10], still has several limitations. When the radionuclide and photosensitizer are administered separately, the CR interaction can be weak and the therapeutic effect of the CR-PDT can be limited [11], while the strategy of coupling a radionuclide with a photosensitizer can lead to the continuous generation of ROS throughout the blood circulation period, making the modality effectively a normal chemotherapeutic agent [12]. Thus, the development of a CR-PDT strategy with high efficacy and tumor accumulation, while minimizing its adverse effects, is of major significance, but remains challenging [12].

Exosomes are considered promising delivery vehicles owing to their excellent biocompatibility, drug-carrying capacity, and tumor targeting ability [13, 14]. However, there are significant barriers to the therapeutic use of exosomes, primarily owing to their low yield from cell cultures [15, 16]. In view of the low yield of exosome production, several studies have demonstrated that exosome-mimetics (EMs) can be used to substitute exosomes [17, 18]. EMs, which are prepared by the serial extrusion of cells, have cell membranes and sizes similar to those of exosomes, higher yield, and the same powerful tumor targeting ability [19–22]. To further improve the efficiency of drug delivery, FDA-approved 5-aminolevulinic acid (ALA) was used to optimize the administration agents. According to FDA data, ALA alone was eliminated quickly after administration, the clinical safety data, which consists of integrated safety data from 527 patients (safety analysis set) who received at least 1 dose of 20 mg/kg, was proved to be safe [23]. And studies have shown that ALA is an extremely powerful drug for PDT [24–26]. In the heme biosynthesis pathway, ALA is a natural precursor of protoporphyrin IX (PpIX), a commonly used photosensitizer in clinical PDT [27]. Abundant mitochondria are necessary for the conversion of ALA to PpIX. Thus, at the tumor site, CR from ^131^I decay activates PpIX to produce ROS, which kill tumor cells. However, in normal tissues, the conversion of ALA to PpIX is limited, resulting in little damage. In addition, the dominant emission of CR is in the ultraviolet and blue regions of the visible spectrum [28], which makes CR ideal for triggering ultraviolet and blue wavelength-responsive PSs such as PpIX [29].

**Scheme 1:**
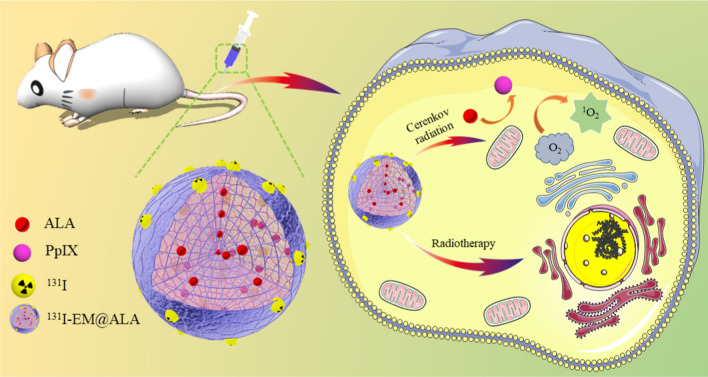
Illustration of the ALA-loaded and ^131^I-labeled exosome-mimetics nanoparticles (^131^I-EM@ALA) used for synergistic internal radiotherapy and Cerenkov radiation induced photodynamic therapy

On the basis of these previous observations, we designed a novel modality with a view to optimizing the therapeutic effect of CR-PDT to reduce side effects and improve the antitumor efficacy of CR-PDT. Efficient labeling of ALA-loaded EMs (EM@ALA) with ^131^I was achieved using the chloramine-T method (^131^I-EM@ALA). ^131^I-EM@ALA was expected to accumulate in tumors following intravenous (iv) administration because of the enhanced permeability and retention (EPR) effect and homologous targeting characteristics [20–22]. The abundant mitochondria at the tumor site were then expected to convert ALA into the active photosensitizer PpIX, which would generate ROS under CR stimulation. The combination of ^131^I radiation therapy (RT) and CR-PDT was therefore expected to achieve a strong antitumor effect, while the relatively low accumulation of PpIX in normal tissues would prevent the release of payload to minimize any adverse effects of CR-PDT. Overall, the local generation of PpIX in tumor tissue was expected to significantly enhance the tumor therapy effect and reduce the adverse effects of CR-PDT, providing a promising strategy for CR-PDT tumor therapy (Scheme 1).

## Materials

### Preparation and characterization of EM@ALA

Experiments were performed with mouse breast cancer cell line 4T1, which was maintained in 1640 (Gibco) containing 10% fetal bovine serum (FBS, Gibco), 10,000 units of penicillin and 10 mg/ml of streptomycin. Cells were cultivated at 37 ℃ and 5% CO_2_. EM@ALA was prepared according to the previous report [30]. 4T1 cancer cell line was a generous gift from Oncology Department, Union Hospital, Tongji Medical College, Huazhong University of Science and Technology. 4T1 cancer cells were collected by trypsin, resuspended by hypotonic solution (PBS: ddH2O = 1:3), and disrupted at 4 ℃ for 2 h. Afterwards, the broken 4T1 cells were centrifuged at 700*g* for 10 min at 4 °C and the collected sediment was resuspended and centrifuged again at 14,000*g* for 30 min. After that, the 4T1 cell membrane and ALA solution were passed through a 0.4um polycarbonate membrane by extruded physically for 11 times.

The result of particle size and zeta potential distributions were gained by using a Zetasizer Nano ZS (Malvern Instruments, Malvern, UK). EM@ALA solutions were stored at 4 ℃ for stability analysis. A transmission electron microscope (TEM; JEM-2010 ES500 W, Japan) operating at 200 keV was used to show the morphology of EM@ALA. Representative protein of EM@ALA was analyzed by western blot (WB). The absorption properties of samples was revealed by UV–vis spectrometry (Synergy 2, BioTek Instruments Inc., USA).

### The preparation of ^131^I-EM@ALA

^131^I-EM@ALA was prepared by chloramine T method [31]. Briefly, 40 μl (30 mci/ml) ^131^I (Gaotong, Chengdu, China) solution was added to 50 μl EM@ALA solution, then 15 μl 25 mg/ml chloramine T solution was added to the mixtures and vortexed for 90 s. Then 5 μl 100 mg/ml sodium pyrosulfite solution was added to terminate the reaction. The labeling efficiency and stability were measured by thin layer chromatography (TLC). The instant thin-layer chromatography-silica gel (iTLC-SG) was used as the medium and normal saline as the developing agent. The stability of labeling rate in fetal bovine serum (FBS) and PBS within 48 h was measured by this method.

### Cell viability experiment

CCK-8 assay was used to evaluate the cytotoxicity effect of ^131^I-EM@ALA. 4T1 cells were seeded in 96-well plates at a density of 6000 cells/well and cultured for 24 h. ^131^I-EM@ALA, ^131^I-EM, EM@ALA, Na^131^I and PBS were added to the medium with different concentrations, and incubated for another 24 h. After incubation, the CCK-8 assay was performed to determine the cell viability.

### Cellular uptake and localization of ^131^I-EM@ALA

4T1 cells were seeded in 24-well plates at a density of 1 × 10^5^ cells/well and cultured for 24 h. Then ^131^I-EM@ALA (2 μci/well) or Na^131^I (2 μci/well) were added to the medium respectively. After incubated for different time points (1, 2, 4, and 6 h), cells and supernatant were collected and measured with a well-type γ-counter (2470 Automatic Gamma Counter WIZARD, PerkinElmer, Norwalk CT, USA).

4T1 cells were seeded into each 24-well plate at a concentration of 6 × 10^4^ cells per well and cultured for 24 h. After being treated with cy5 labeled EM@ALA for different time points, the tumor cells were observed by using a fluorescence confocal microscope or analyzed by flow cytometry.

### Cerenkov radiation with ^131^I Radioisotope

Cerenkov radiation was detected using an IVIS system (PerkinElmer Inc.) under various conditions (PBS, ALA, PpIX, Na^131^I, Na ^131^I + ALA, and Na ^131^I + PpIX, all the drugs were free condition). The images were acquired with different emission filters, and the excitation was blocked.

### Western blot

4T1 cells were seeded in 6-well plates at a density of 3 × 10^6^ cells/well and cultured for 24 h. Then PBS, EM@ALA, ^131^I-EM, ^131^I-EM@ALA were added to the medium respectively and cultivated for 24 h. Total protein was gained from 4T1 cells by RIPA buffer (Beyotime Biotechnology, China). The tumor-bearing mice were injected PBS, EM@ALA, ^131^I-EM (0.2 mci), ^131^I-EM@ALA (0.2 mci) respectively. Total protein was gained from the tumor of mice by RIPA buffer (Beyotime Biotechnology, China). The protein concentrations of tumor cells were measured with BCA protein Assay Kit (Beyotime Biotechnology, China). Equal amounts of protein (20 μg) were added into each lane of a 10% SDS-PAGE (New Cell & Molecular Biotech, China) and separated. Then transferred the protein onto PVDF membranes (Millipore, United States), and the membranes were blocked by Protein Free Rapid Blocking Buffer (EpiZyme, China) for 2 h and incubated with primary antibodies at 4 ℃ for 12 h. The primary antibodies used are as followed: anti-Caspase 3 (1:1000, Abclonal, China), anti-γ-H2AX (1:1000, Proteintech, United States), anti-KU70 (1:5000, Proteintech, United States), anti-RAD51 (1:1000, Proteintech, United States), and anti-GAPDH (1:1000, Abclonal, China). After incubation with HRP-coupled secondary antibody (goat anti-rabbit or rabbit anti-mouse IgG, 1:10,000, Boster, China), the membranes were treated with chemiluminescence (Solarbio, China) for 3 min and visualized on a Visionwork system.

### Animal models

All animal studies were conducted under the guidance and approved by the Institutional Animal Care and Use Committee of Tongji Medical College of Huazhong University of Science and Technology. BALB/c mice (female, 5–6 weeks old) were purchased from Weitong Lihua Laboratory Animal Center (Beijing, China), and maintained in a pathogen-free environment. 4T1 cells (1 × 10^7^ in 100 µl PBS) were subcutaneously injected into the right front leg of the BALB/C mice (Weitong Lihua Laboratory Animal Center, Beijing, China). The tumor-bearing mice were used for the following experiments after the tumor volume reached approximately 80 mm^3^. The tumor volume was calculated with the formula: length × width^2^ × 0.5.

### In vivo fluorescence imaging

When the tumor volumes ranged between 80 and 100 mm^3^, ICG-EM@ALA was injected to the tumor-bearing mice. The in vivo fluorescence imaging was conducted at different time points after injection. Tumors and organs of interest were resected and imaged 24 and 48 h later.

To detect the fluorescence signal of PpIX in vivo, 4T1 tumor bearing mice were sacrificed, and the interested tissues (e.g., heart, liver, kidneys, muscle, and tumor) were harvested for the fluorescence detection of PpIX by animal fluorescence imaging in 24 h after EM@ALA injection.

### SPECT/CT imaging and biodistribution experiment

Single-Photon Emission Computed Tomography/Computed Tomography (SPECT/CT) imaging was conducted to observe the whole-body distribution of ^131^I-EM@ALA in 4T1 tumor-bearing mice using a human SPECT/CT device (NM670, GE, United States). Four days before the SPECT/CT imaging experiment, all mice were fed with 1‰ potassium iodide solution. The mice of experimental group were injected with ^131^I-EM@ALA (0.2 mCi, 0.1 ml) while the mice of control group were injected with Na^131^I (0.2 mCi, 0.1 ml). The SPECT/CT images were acquired 24 h after the drug injection.

For the biodistribution experiment, 4T1 tumor-bearing mice (n = 4 in each group) were injected with ^131^I-EM@ALA or Na^131^I (0.2 mCi, 0.1 ml) via tail vein, and sacrificed at 24 h. The organs (e.g., blood, heart, liver, muscle, tumor) of interest were harvested and weighed. Radioactivity of tissue was quantified using a γ-counter.

### Therapeutic effect in animals

To explore the ^131^I-EM@ALA treatment effect, the mice were divided into 4 groups (n = 8 per group) randomly for various treatments when the tumor volumes reached 80 mm^3^: (1) PBS; (2) EM@ALA; (3) ^131^I-EM; (4) ^131^I-EM@ALA. The dosages of ^131^I and ALA in these studies were 25 μCi/g and 30 μg/g, respectively. Tumor volume was measured once every 2 days for 19 days. The survival rate was assessed by the Kaplan–Meier method. The end point events were defined as follow: tumor volumes > 1500 mm^3^; ulcerating tumor tissue; mortality; > 15% weight loss.

### In vivo toxicity evaluation

Group division and drugs injection were as described above (n = 4 per group). In vivo toxicity was assessed using body weight change, hematoxylin and eosin (H&E) staining, hematology and blood biochemistry indices. The general state of the animals was observed daily, and the body weight of the mice was monitored every other day. At the end of the 19-day treatment, whole blood was collected and analyzed to determine aspartate aminotransferase (AST), alanine aminotransferase (ALT), blood urea nitrogen (BUN), creatinine (CRE), and alkaline phosphatase (ALP) using a biochemical analyzer (Chemray240, China). Meanwhile, the samples of blood and interested tissues (heart, lung, liver, kidney, spleen, muscle, and thyroid) were harvested for H&E staining. Besides, since we are interested in the toxicity differences between ^131^I-EM@ALA group and ^131^I-EM@PpIX group, tumor-bearing mice were sacrificed after 7, 14 and 21 days from the nanoparticle application, and samples of blood and interested tissues were harvested for the further analysis.

### Histological analysis

Interested organs and tumor tissues were taken out from mice and fixed with 4% paraformaldehyde, then embedded in paraffin after 19-day treatment. The sliced interested organs and tumor tissues were further performed for H&E, terminal deoxynucleotidyl transferase-mediated dUTP-biotin nick end-labeling (TUNEL) and Ki67 staining.

### Statistical analysis

All values were presented as the means ± standard deviation (SD). The significance level was set as 0.05, and the data were presented with (*) for p < 0.05, (**) for p < 0.01, and (***) for p < 0.001, respectively.

## Results

### Fabrication and characterization of EM@ALA

**Fig. 1:**
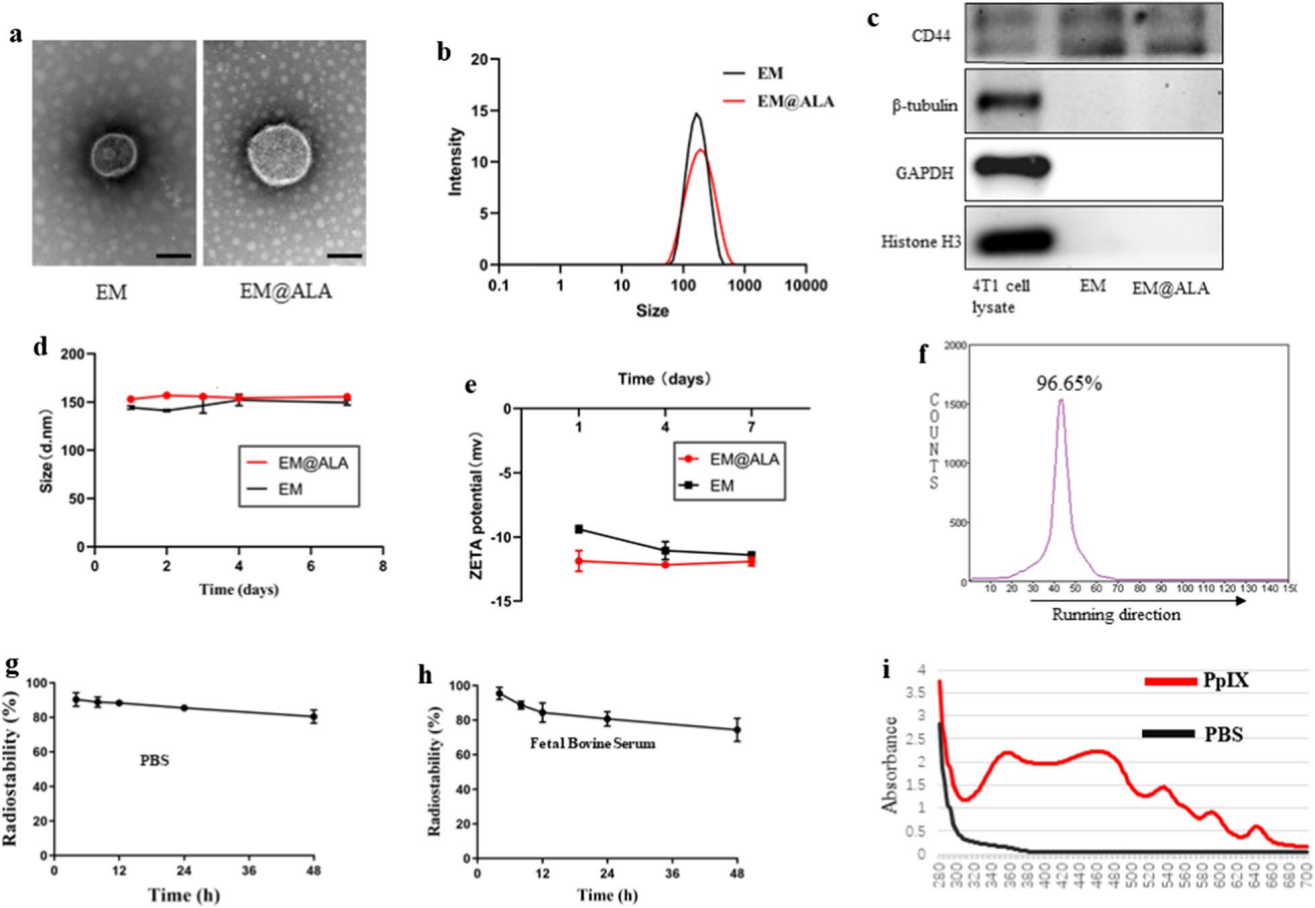
Characteristics of ^131^I-EM@ALA. 4T1 tumor cells derived drug-loaded EM and empty EM assessed by TEM (**a**), DLS (**b**) and WB (**c**). Scale bar = 100 nm. Size (**d**) and Zeta potential (**e**) stability test of EM@ALA and EM in PBS for 7 days. **f** The representative radiochemical purity of ^131^I-EM@ALA. Radiolabeling stability test in PBS (**g**) and FBS (**h**) during 48 h (n = 3). **i** UV–vis spectrum of PpIX and PBS

Transmission electron microscopy (TEM) results showed the spherical morphology of EM@ALA and EM nanoparticles (Fig. 1a). Dynamic light scattering (DLS) experiments demonstrated that the mean radius of the EM@ALA and EM nanoparticles was 154.8 ± 2.8 nm and 144.4 ± 1.4 nm, respectively (Fig. 1b). To determine whether functionalized membrane proteins were retained on the EM@ALA and EM, the protein content of the EM@ALA and EM particles was analyzed by western blot. Compared with the 4T1 cell lysate, the cell membrane proteins were mostly retained in the form of EM@ALA and EM (Additional file 1: Fig. S2). The results showed that EM@ALA and EM presented similar protein profiles to that of 4T1 cell lysate (Fig. 1c). The membrane-specific markers in EM@ALA and EM, such as positive antigen CD44, were well inherited from 4T1 cells, while the cytosol marker (i.e., glyceraldehyde 3‑phosphate dehydrogenase (GAPDH)) and the nuclear protein marker (i.e., histone H3) were almost undetected in the final EM@ALA and EM particles. In addition, the size of EM@ALA changed slightly from 153.1 ± 3.0 nm on the first day to 155.5 ± 2.2 nm on the seventh day; The Zeta potential of EM@ALA also changed slightly from − 11.8 ± 0.7 mv on the first day to − 11.9 ± 0.3 on the seventh day, indicating the excellent stability of the nanoplatform (Fig. 1d, e). The radiolabeling efficiency to give ^131^I-EM@ALA was approximately 96.65% in PBS (Fig. 1f). The radiochemical stabilities were 80.5 ± 1.3% in PBS and 74.3 ± 2.2% in FBS for up to 48 h (Fig. 1g, h), which suggests that the radiolabeled ^131^I-EM@ALA nanoplatform had excellent biological stability in terms of size and radiochemistry profile and could be applied to in vivo biological systems. In further experiments (Fig. 1i), ALA, which can be transformed into PpIX in mitochondria, was found to be the optimal PS because PpIX has an ideally matched absorbance wavelength (λ_abs_ < 480 nm) for Cerenkov light (major emission in the ultraviolet and blue regions of the visible spectrum [28], λ < 480 nm).

### Cellular uptake and effects of ^131^I-EM@ALA treatment

**Fig. 2:**
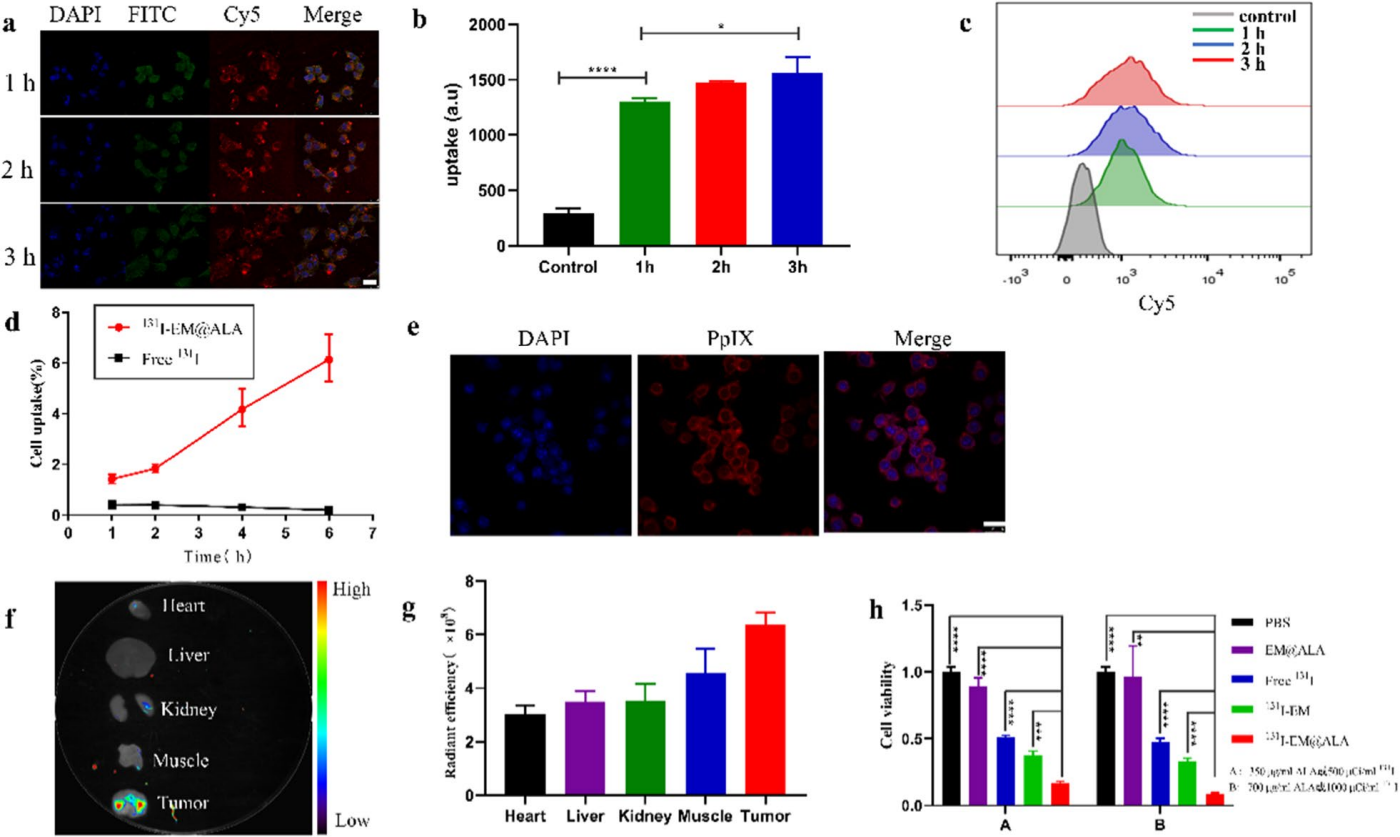
Tumor-binding and antitumor effects of ^131^I-EM@ALA. Confocal fluorescence images (**a**) and flow cytometry (**b**, **c**) of tumor cells after incubation with Cy5@EM@ALA for different times. Scale bar = 25 μm. Cell nuclei were stained blue with DAPI, filamentous actin cytoskeletons were stained green with FITC phalloidin. **d** In vitro uptake of ^131^I labeled EM@ALA or Na^131^I by 4T1 cells at different points. **e** EM@ALA and 4T1 cells were incubated for 2 h before visualization of PpIX-specific fluorescence by confocal microscopy. Scale bar = 25 μm. 24 h after EM@ALA injection, ex vivo fluorescence images (**f**) of PpIX signal and semi-quantitative analysis (**g**). **h** Viability of 4T1 cells with various treatments

Cy5-labeled EM@ALA nanoparticles were incubated with 4T1 cells for different time periods and the results were analyzed by confocal laser scanning microscopy and flow cytometry. Confocal images revealed strong Cy5-red fluorescence signals in the cytoplasm at 1, 2, and 3 h (Fig. 2a). Flow cytometry analysis (Fig. 2b and c) showed that the tumor cell binding increased slightly over time, and there was no statistical difference between the 1-h incubation and 2-h incubation samples (p = 0.13), or the 2-h incubation and 3-h incubation samples (p = 0.08); however, a statistical difference was observed between the 1-h incubation and 3-h incubation samples (p < 0.05). To accurately determine the uptake rate, we performed a cell uptake assay using a radionuclide label. As illustrated in Fig. 2d, the uptake of ^131^I-EM@ALA by 4T1 cells increased gradually over time and reached a maximum at 6 h, which was significantly higher than the uptake of Na^131^I (p < 0.001).

We then verified the ability of ALA-derived PpIX to accumulate in cancer cells. Twenty-four hours after intravenous injection, a red fluorescence signal from PpIX in 4T1 cells was clearly detected (Fig. 2e). Subsequently, we examined the conversion of ALA to PpIX in vivo. PpIX fluorescence signals in the tumors and major organs showed that ALA was successfully converted to PpIX—predominantly within tumors (by endogenous enzymes)—and little PpIX accumulated in the liver (Fig. 2f). Semi-quantitative analysis showed that the PpIX fluorescence intensity in tumors was approximately 1.8-fold that in the liver (Fig. 2g). As illustrated in Fig. 2h, the relative viabilities of the 4T1 cells had a negative correlation with both the ALA concentration and ^131^I activity. In the absence of laser light, EM@ALA exhibited no clear cytotoxicity towards 4T1 cells at two different concentrations. At an ALA concentration of 700 μg/ml and ^131^I radioactivity of 1000 μCi/ml, the viability of the 4T1 cells was 100 ± 3%, 96.8 ± 18.5%, 47.3 ± 2.4%, 33.1 ± 1.9%, and 8.8 ± 0.6% following treatment with PBS, EM@ALA, Na^131^I, ^131^I-EM, and ^131^I-EM@ALA, respectively. The ^131^I-EM@ALA group showed the best antitumor effect of groups tested, demonstrating the cooperative effect of radiotherapy and CR-PDT. In addition, ^131^I-EM showed a better treatment effect than Na^131^I, which may be because of the higher cell uptake of ^131^I-EM.

### Cerenkov radiation imaging

**Fig. 3:**
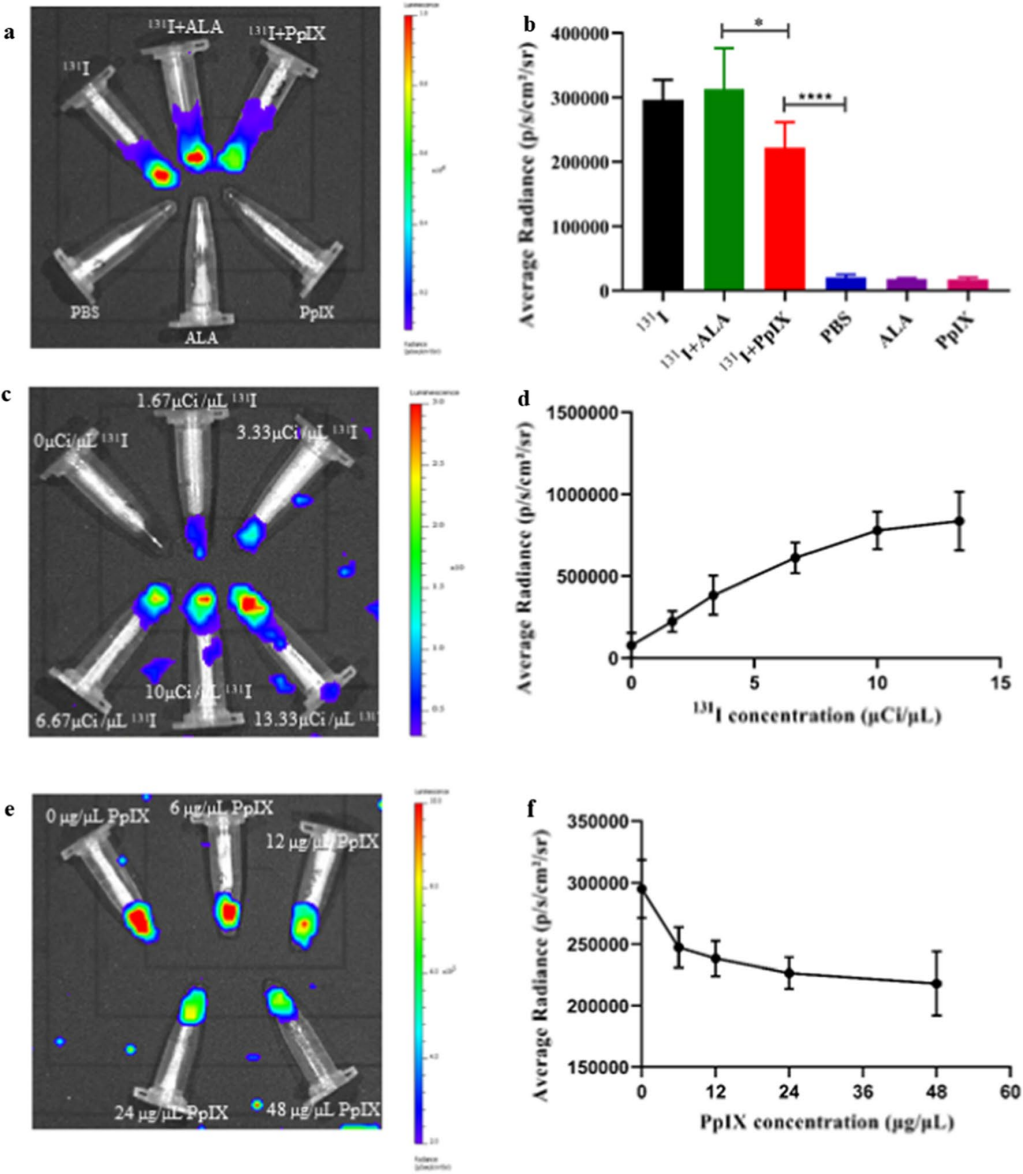
Cerenkov radiation. **a** Cerenkov radiation imaging in different kinds of solutions and the semi-quantitative analysis (**b**). **c** Cerenkov radiation imaging with 1.5 μg/μl PpIX and different concentration of ^131^I and the semi-quantitative analysis (**d**). **e** Cerenkov radiation imaging with 4 μCi/μl ^131^I and different concentration of PpIX and the semi-quantitative analysis (**f**)

To detect the Cerenkov radiation and CRET, we used a small-animal in vivo imaging system (IVIS) to collect ^131^I emission without excitation light. As shown in Fig. 3a and b, the solutions containing PBS, ALA, and PpIX did not emit fluorescence, while we detected a clear fluorescence signal from the solutions containing ^131^I, ^131^I plus ALA, and ^131^I plus PpIX, and the signal of the ^131^I plus PpIX solution was higher than that of the ^131^I plus ALA solution (p < 0.05). The fluorescence signals of the solutions containing ^131^I and PpIX increased when the concentration of ^131^I increased (Fig. 3c and 3d), as did those of the solutions containing ^131^I without PpIX (Additional file 1: Fig. S3), while the fluorescence signal decreased when the concentration of PpIX increased (Fig. 3e and f). These results indicate that the CR energy could be transferred to PpIX.

### In vivo NIRF imaging

**Fig. 4:**
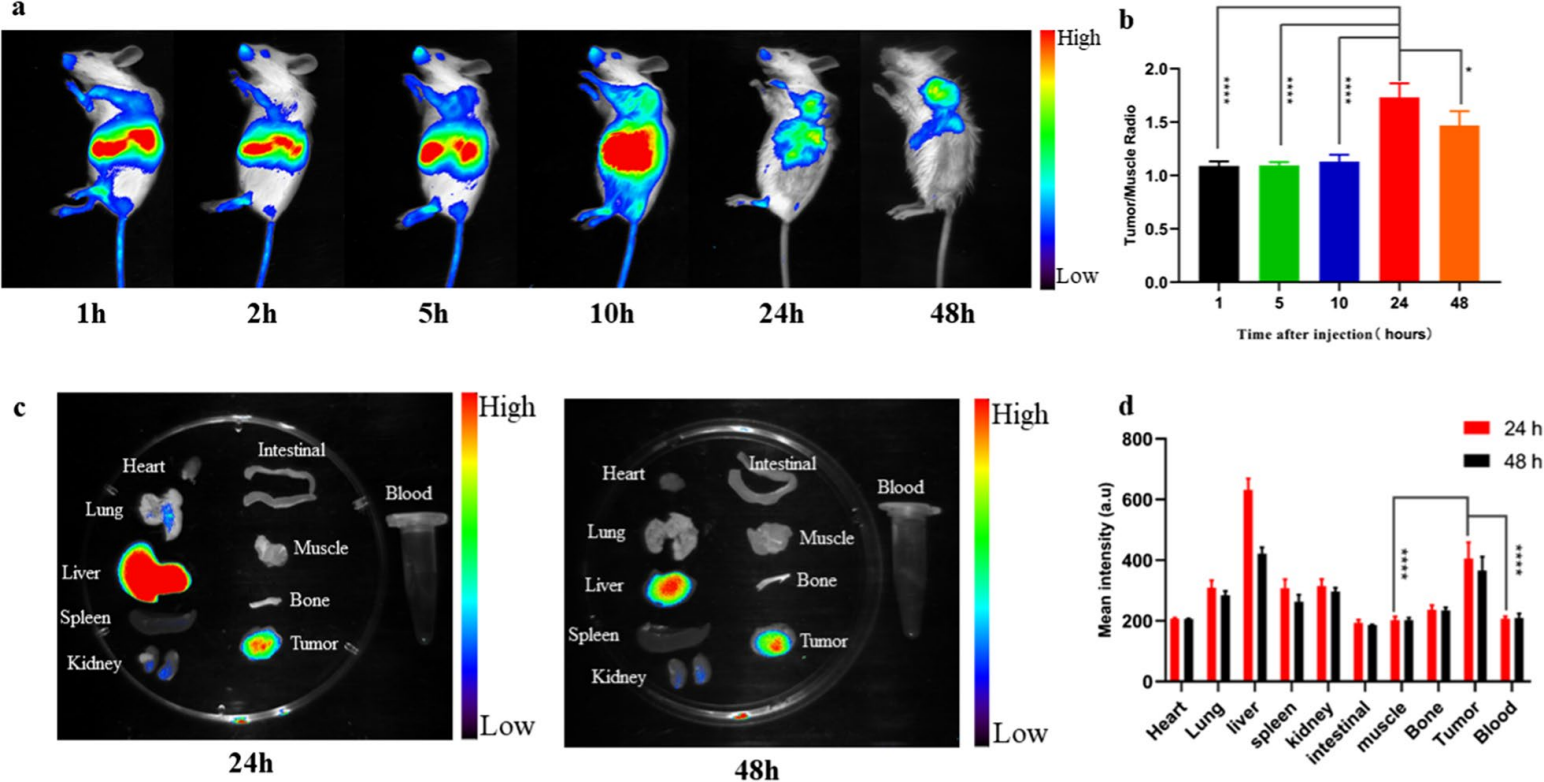
In vivo and ex vivo imaging with ICG-EM@ALA. In vivo fluorescence images (**a**) of 4T1 tumor-bearing mice taken at different time points and semi-quantitative analysis. **b** Ex vivo fluorescence images (**c**) of major organs and tumor dissected from mice at 24 h and 48 h and Semi-quantitative analysis (**d**)

The distribution of the nanoparticles in vivo was investigated by intravenously injecting ICG-EM@ALA into 4T1 tumor-bearing mice. The in vivo fluorescence signals in the tumors gradually increased over time and reached maximum intensity at 24 h, suggesting time-dependent accumulation of ICG-labeled EM@ALA in the tumor (Fig. 4a). The T/M ratio peaked at 24 h after injection (1.7 ± 0.1; Fig. 4b). The ex vivo fluorescence images further confirmed the accumulation of ICG-EM@ALA at the tumor site. Notably, strong fluorescence was also detected in the liver, likely due to the clearance of EM@ALA nanoparticles by the liver (Fig. 4c and d).

To further confirm the accumulation of ^131^I-EM@ALA at the tumor sites, SPECT/CT imaging was carried out to evaluate the distribution of ^131^I-EM@ALA in vivo. SPECT/CT images were acquired 24 h after injection. The signal in the tumor region was markedly higher than that in surrounding tissues (Additional file 1: Fig. S3). The biodistribution results showed that the T/B (tumor to blood) and T/M (tumor to muscle) ratios were approximately 2.1 ± 0.6 and 4.7 ± 1.1, respectively (Additional file 1: Fig. S3). The results of imaging and biodistribution showed that ^131^I-EM@ALA effectively accumulated in the tumor region.

### In vivo tumor therapy in a 4T1 tumor-bearing mouse model

**Fig. 5:**
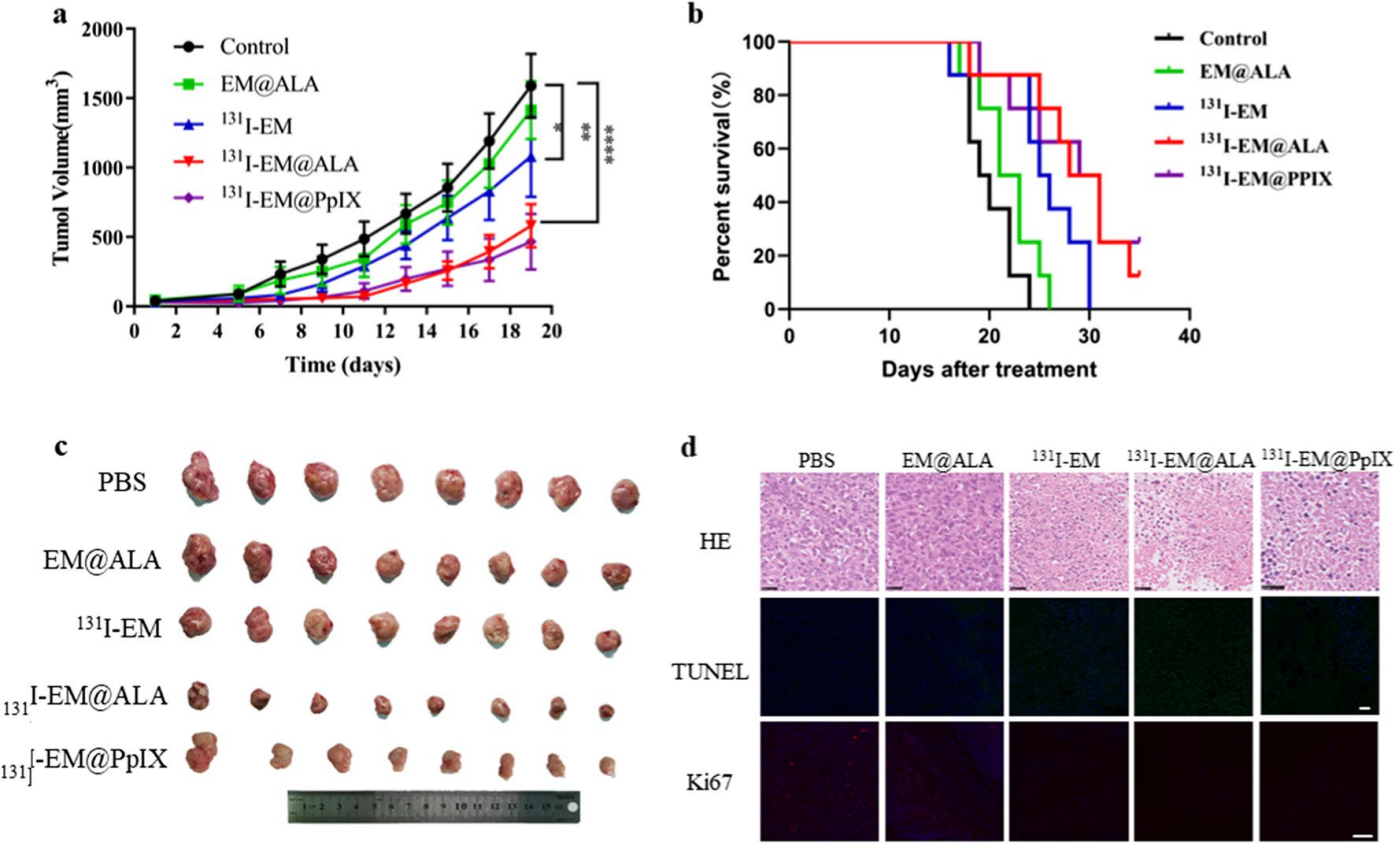
In vivo antitumor effects of ^131^I-EM@ALA. The tumor growth (**a**) and survival curves (**b**) of 4T1 tumors after different treatments. **c** Photographs of excised tumors at day 19. **d** HE staining of tumors. Scale bar = 25 μm. Ki67 staining and TENEL staining. Scale bar = 100 μm

We examined whether the combination of CR-PDT and radiotherapy could effectively inhibit tumor growth. At 19 days post treatment, the tumor volume for the ^131^I-EM@ALA group (580.0 ± 146.4 mm^3^) was significantly smaller than those of the ^131^I-EM (1081.5 ± 273.5 mm^3^) or EM@ALA groups (1414.9 ± 194.9 mm^3^), which indicated that combination therapy had greater antitumor efficacy than ^131^I-radiotherapy alone (Fig. 5a). Notably, no statistical difference was found between the EM@ALA group and control group, indicating that ^131^I CR was critical to CR-PDT. The survival rates were recorded over 35 days (Fig. 5b). ^131^I-EM@ALA (12.5%, 35 days) and ^131^I-EM@PpIX (25%, 35 days) markedly prolonged the survival time compared with the control (0%, 24 days). At the end of the treatments, the tumors were harvested (Fig. 5c) and stained (Fig. 5d). H&E staining of the tumor tissues showed reduced nucleus-to-cytoplasm ratios, which were related to a decrease in the number of cancer cells in mice injected with ^131^I-EM@ALA. Ki67 staining and TUNEL staining showed that treatment with ^131^I-EM@ALA and ^131^I-EM@PpIX induced the highest rate of apoptosis and an optimal level of necrotic lesions.

### In vivo toxicity evaluation

**Fig. 6:**
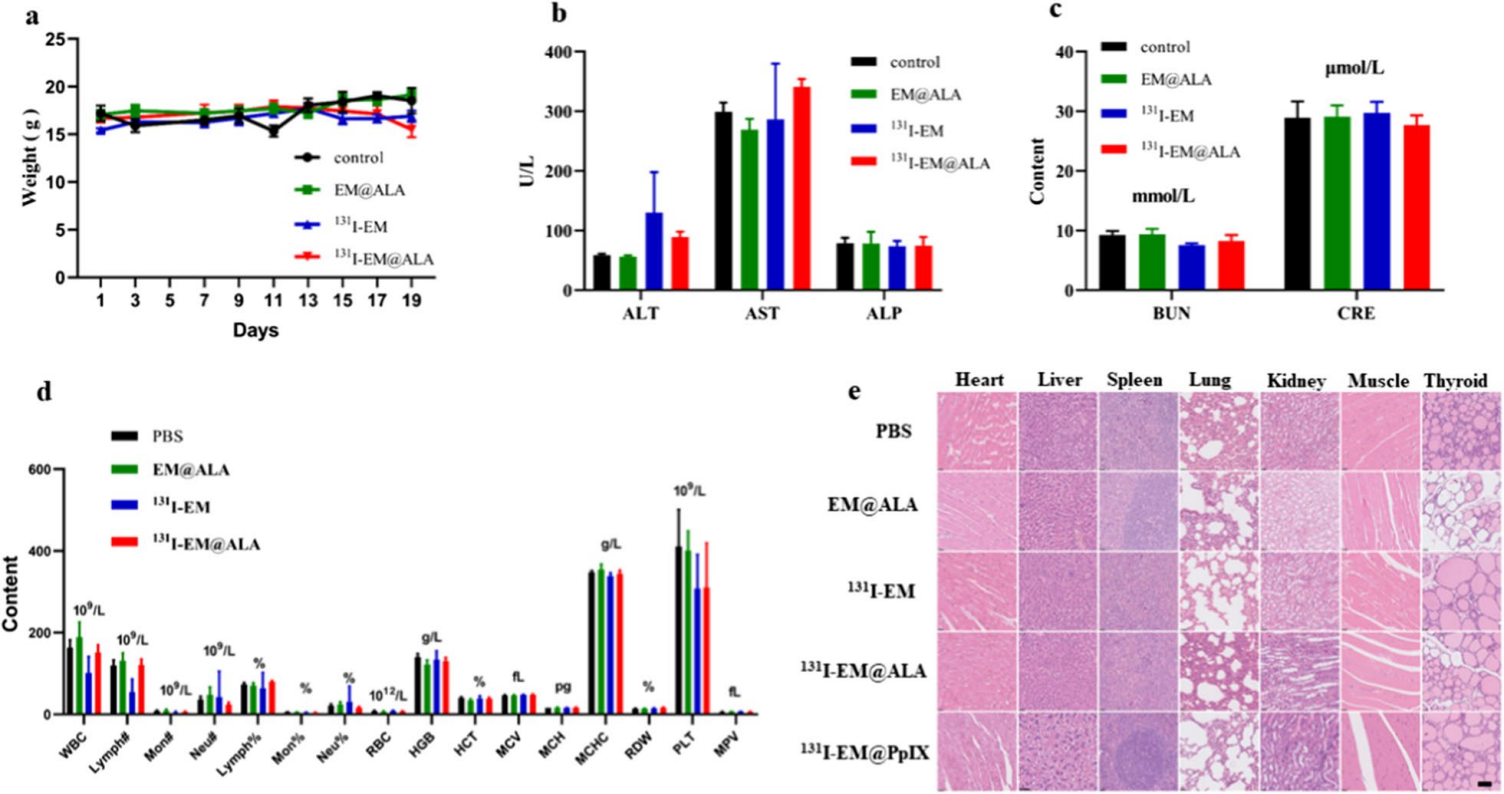
In vivo toxicity test. **a** Changes in animal body weight at 19 days. Blood biochemistry data including liver function markers **b**: alanine aminotransferase (ALT), aspartate aminotransferase (AST) and alkaline phosphatase (ALP) and kidney function markers **c**: blood urea nitrogen (BUN), creatinine (CRE). **d** Blood routine parameters data. White blood cells (WBC), lymphocytes number (Lymph#), monocyte number (Mon#), Neutrophil number (Neu#), lymphocytes percentage (Lymph%), monocyte percentage (Mon%), neutrophil percentage (Neu%), red blood cells (RBC), hemoglobin (HGB), hematocrit (HCT), mean corpuscular volume (MCV), mean corpuscular hemoglobin (MCH), and mean corpuscular hemoglobin concentration (MCHC), red cell volume distribution width (RDW), Platelets (PLT), mean platelet volume (MPV). **d** HE-stained slice images of major organs. Scale bar = 50 μm

No obvious weight loss was observed for any of the groups during the 19-day experiment (Fig. 6a). The results showed that both routine blood parameters and blood biochemistry parameters were within the normal ranges of fluctuation (Fig. 6b–d). Representative H&E staining showed that there was no obvious histological damage (Fig. 6e). These results suggest that ^131^I-EM@ALA had excellent biocompatibility.

**Fig. 7:**
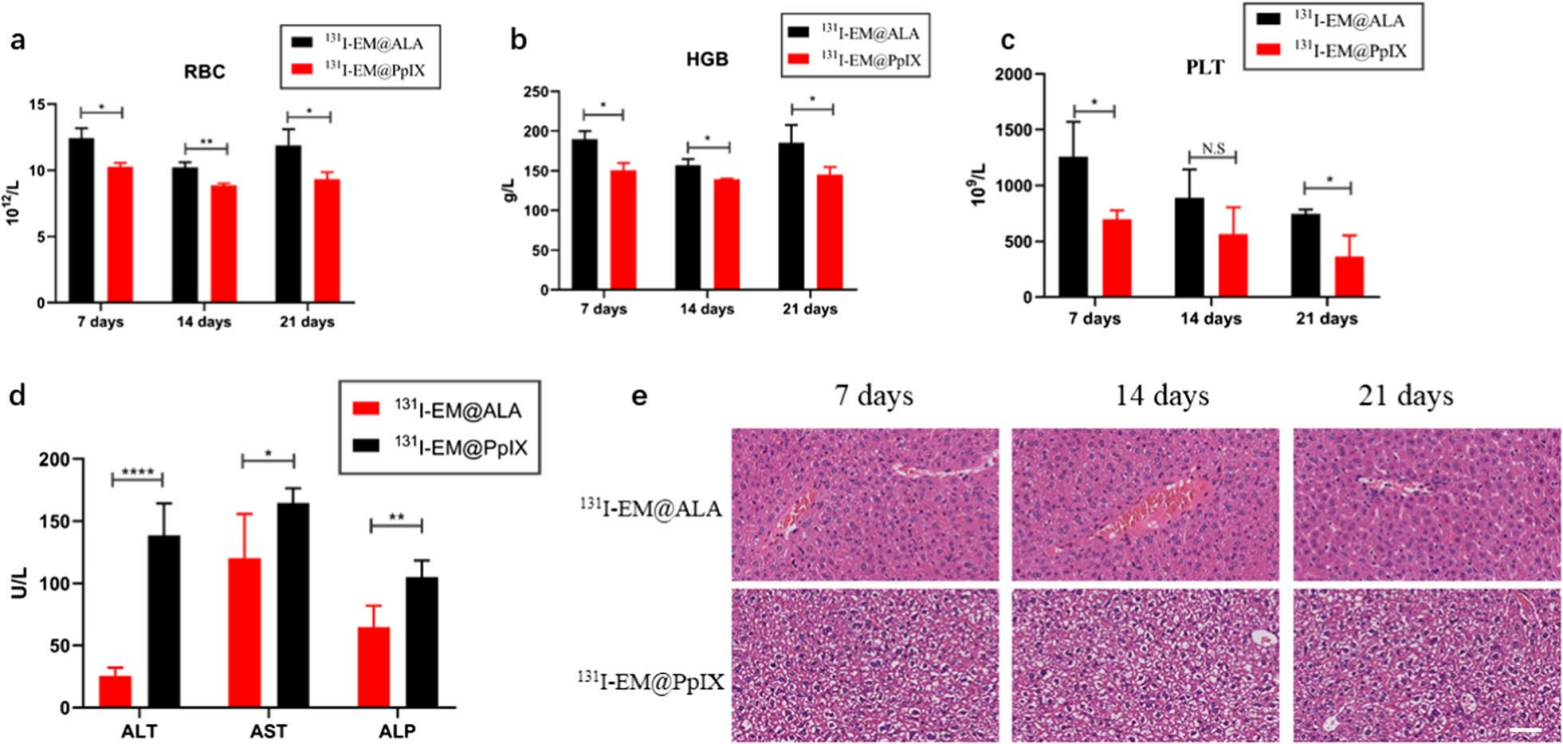
Toxicity comparison of two groups over time. **a**–**c** RBC, PLT and HGB levels at 7, 14, and 21 days. **d** AST, ALT, and ALP levels at 21 days. **e** HE-stained tissues from mice to monitor the histological changes in liver at different time points after intravenous injection of ^131^I-EM@ALA and ^131^I-EM@PpIX. Scale bar = 100 μm

We compared the effects of ^131^I-EM@ALA and ^131^I-EM@PpIX in vivo to evaluate the potential of our strategy to reduce toxicity. As shown in Fig. 7a–c, the RBC, PLT, and HGB of the ^131^I-EM@PpIX group were lower than those of the ^131^I-EM@ALA group at 7, 14, and 21 days (p < 0.05), except for PLT at 14 days (p = 0.18), indicating that the blood cells in the ^131^I-EM@PpIX group were impaired. At 21 days post treatment, the levels of AST, ALT, and ALP for the ^131^I-EM@PpIX group were significantly higher than those of the ^131^I-EM@ALA group (Fig. 7d), indicating that hepatocytes were damaged by ^131^I-EM@PpIX. H&E staining at 7, 14, and 21 days showed severe oedema for hepatocytes in the ^131^I-EM@PpIX group, but not in the ^131^I-EM@ALA group (Fig. 7e), indicating marked damage to liver cells in the ^131^I-EM@PpIX group. We therefore inferred that the damage was caused by CR-PDT, but not radiotherapy. All the results show that our strategy of using a photosensitizer precursor (ALA) instead of photosensitizers (such as PpIX) greatly reduced the adverse effects.

### Tumor cell apoptosis mechanism

**Fig. 8:**
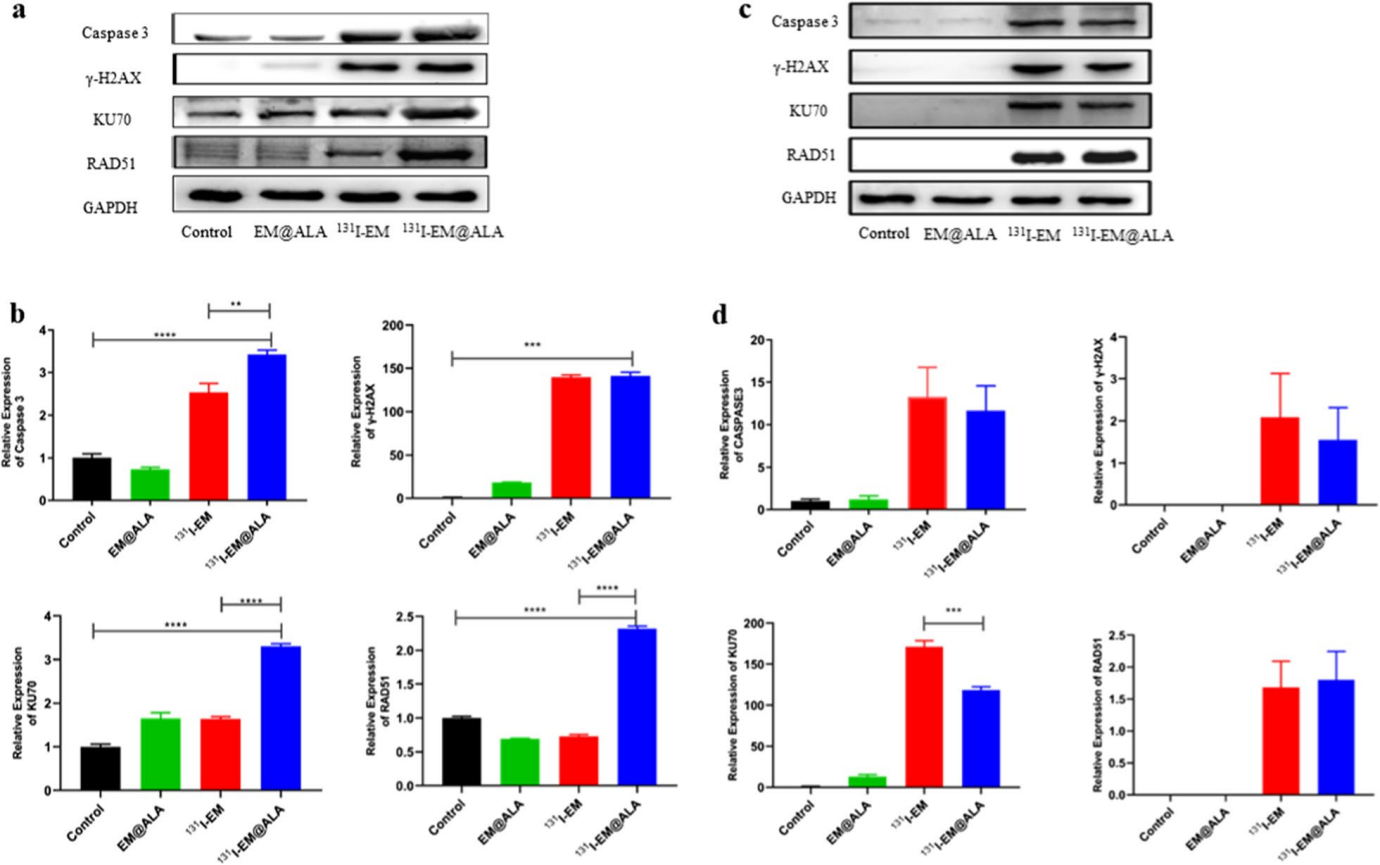
Effect of ^131^I-EM@ALA treatment on apoptotic-related proteins (caspase 3, γ-H2AX, KU70, and RAD51) in 4T1 cells determined by western blot in vitro (**a**) and in vivo (**c**). The relative protein expressions of caspase 3, γ-H2AX, KU70, and RAD51. The protein expression levels were detected and evaluated in vitro (**b**) and in vivo (**d**) by Image J software, GAPDH was detected as a loading control

Many recent studies have shown that PDT leads to tumor death via the lysosomal pathway [32, 33]. The in vitro expression of apoptosis related proteins (caspase 3) and radiological DNA damage and repair related proteins (γ-H2AX, KU70, and RAD51) [34–37] was investigated, as shown in Fig. 8a and b. The expression of caspase 3 in the ^131^I-EM@ALA group was significantly higher than those for the ^131^I-EM group (p < 0.01) and control group (p < 0.001). Similarly, the expression of radiological DNA damage and repair related proteins (KU70 and RAD51) for the ^131^I-EM@ALA group was higher than that for the ^131^I-EM group (p < 0.001) and control group (p < 0.001). In the in vivo experiment, the expression of KU70 in the ^131^I-EM@ALA group was lower than that for the ^131^I-EM@ group (Fig. 8c and d).

## Discussion

We designed exosome-mimetic nanoparticles loaded with ALA and modified them with the radionuclide ^131^I (^131^I-EM@ALA). ^131^I-EM@ALA can be accumulated at tumor sites by EPR effect due to the nanoscale size [38]. Besides, because of the plasma membrane proteins on the surface including galectin-3, N-cadherin, and epithelial cell adhesion molecule, tumor-derived micro-vesicles possess some unique properties such as antigenic display and homologous binding [39, 40]. For instance, cancer cell membranes carrying the tumor-specific surface antigens have the excellent homologous selectivity to target the tumor with the same source [20, 41–43]. Studies have shown that exosomes could specifically target receptor cells to deliver their cargoes [44–46]. The homologous targeting of cancer cell membranes has been widely applied in tumor therapy, but the tumor-targeting mechanism is still unclear need to be further elucidated [47]. In our study, the nanoparticles were shown to be stable in structure and have excellent antitumor effects and no obvious side effects. The strategy of using CR to excite the photosensitizer PpIX, which was produced from ALA, effectively suppressed the tumor growth and minimized the side effects on normal organs, particularly the liver and blood cells. This observation is attributed to the conversion of ALA to PpIX mainly occurring at tumor sites and rarely in normal tissue. Our study offers a new therapeutic avenue for PDT that does not rely on external light sources and minimizes side effects.

Biomimetic nanoparticles encapsulated by an active cell membrane are attracting increasing attention [48, 49]. Owing to their preserved cell membrane structure, biomimetic nanoparticles can exhibit special functions, such as extended blood circulation, immune system evasion, and efficient drug delivery, and can be used as vaccines [50, 51]. Cancer cells can form intercellular homologous junctions with membrane proteins because of the surface adhesion molecules expressed on cancer cells. Cancer cell membrane-coated nanoparticles are therefore expected to offer homologous targeting ability, making them the ideal vehicle for drug delivery and effective cancer therapy. Our findings supported this expectation: ^131^I-EM@ALA effectively targeted tumor cells both in vitro and in vivo (Figs. 2a, and 4a). In addition, the results of DLS, TEM, and WB showed that the properties of the exosome-mimetic nanoparticles did not change significantly after modification. The radiolabeling stability was 80.5 ± 1.3% in PBS and 70.4 ± 2.2% in FBS at 48 h, which was sufficient for the subsequent studies. The CR imaging and CRET imaging experiments showed that our CR-PDT strategy had a clear dose-dependent effect (Fig. 3a–f), and the results demonstrated that PpIX could be excited by the UV/blue CL of the radionuclide ^131^I, which in turn induced PDT.

We next verified the synergistic therapeutic effects of radiotherapy and CR-PDT in 4T1 tumor-bearing mice. Cell viability was evaluated and showed that the ^131^I-EM@ALA group had a greater antitumor effect than the ^131^I-EM and EM@ALA groups, indicating that both ^131^I and ALA are required for CR-PDT. In the subsequent animal experiments, the combined radio-photodynamic therapy in the ^131^I-EM@ALA group markedly inhibited tumor growth and prolonged the survival time. The results of H&E staining, Ki67 staining, and TUNEL staining showed the highest rate of apoptosis for ^131^I-EM@ALA. Notably, in our CR-PDT antitumor study, substitution of the photosensitizer PpIX with the photosensitizer precursor ALA did not significantly reduce the efficacy of antitumor therapy.

Ni et al. designed magnetic targeting nanostructures with surface conjugating photosensitizer and chelator-free labeling of ^89^Zr (^89^Zr-MNP/TCPP) for magnetism-enhanced CR-induced PDT, the nanoparticles exhibited high antitumor effect with suppressed tumor growth. But the liver was damaged at 7 days p.i. even through self-recovered within two months. The spatiotemporal colocalization of the photosensitizer with the radionuclide in these studies resulted in excellent therapeutic efficacy, but potential side effects to healthy tissues arouse at the same time. Thus, minimizing side-effects of CR-PDT is important but remains challenging [3, 12]. To optimize the treatment strategy, we designed transformable photosensitizer-loaded nanovesicles and labeled with ^131^I for minimizing side-effects of CR-PDT. Transformable photosensitizer ALA makes our system valuable because ALA converts to the PpIX mainly in cancer cells and PDT activated only at the tumor site. Subsequently, we assessed the side effects of combined radio-photodynamic therapy. The strategy of coupling a radionuclide with a photosensitizer has the potential to continuously generate ROS during blood circulation, effectively creating a normal chemotherapeutic agent, which has been shown to result in side effects to healthy tissues [12]. In this study, we designed a nanoparticle loaded with ALA and labeled with ^131^I. At the tumor site, ALA is converted to the photosensitizer PpIX in the abundant mitochondria, then ^131^I-derived Cerenkov radiation combined with the PpIX activates CR-PDT, leading to tumor death. In contrast, in normal tissues, ALA is rarely converted to PpIX; therefore, PDT is not activated, leading to limited side effects on normal tissues. Following the 19-day treatment, no weight loss was observed, and the routine blood parameters, blood biochemistry parameters, and H&E staining were within the normal range of fluctuation for the ^131^I-EM@ALA group; however, liver damage was clearly observed in the ^131^I-EM@PpIX group (Fig. 6a–e). We therefore compared the effects of ^131^I-EM@ALA and ^131^I-EM@PpIX by monitoring the levels of RBC, HGB, and PLT and liver H&E staining every 7 days. The results showed that ^131^I-EM@PpIX caused blood cell and hepatocyte damage that was not observed for the ^131^I-EM@ALA group (Fig. 7a–e). A possible reason for this may be that ^131^I-EM@PpIX continuously produced ROS during its blood circulation, particularly in the blood and liver (the EM nanoparticles were found to accumulate in the liver, Fig. 4a).

To further clarify the molecular mechanism of ^131^I-EM@ALA for synergistic internal radiotherapy and Cerenkov radiation, we tested the apoptotic-related proteins in liver and autophagy-related proteins (P62 and LC3) in 4T1 cell and 4T1 tumor tissue. The results showed that, radiological DNA damage and repair related proteins (KU70 and RAD51) for the ^131^I-EM@ALA group were overexpressed both in liver and tumor cells. Compared with the control group, the expression of caspase 3 in liver did not change significantly in ^131^I-EM@ALA group, which means ROS-mediated apoptosis did not occur in liver (Additional file 1: Fig. S6a). Besides, ^131^I-EM@ALA significantly reduced protein expressions of P62 and increased protein expressions of LC3 (Additional file 1: Fig. S6b and c). In the autophagy process, LC3 will overexpress while the autophagy substrate p62 will be specifically degraded [52]. These results indicated that the autophagy level was significantly elevated in the ^131^I-EM@ALA group compared to the controls. Studies showed that ALA-mediated PDT could induce superoxide anion-dependent autophagic cell death [53], which is consistent with our findings. Thus, we inferred that both radiation injury and ALA-mediated PDT were the molecular mechanism of ^131^I-EM@ALA combination therapy, but ALA-mediated PDT was the dominant one.

## Conclusion

The multifunctional platform ^131^I-EM@ALA was successfully synthesized for the effective delivery of ^131^I and ALA. By extending the blood circulation, ^131^I-EM@ALA improved the delivery of ^131^I and ALA into cancer cells both in vitro and in vivo, thereby enhancing the therapeutic effect. The ^131^I not only acts as a radiotherapeutic, but its CR also provides an internal light source. The results showed that the subcutaneous tumor-bearing mice could be significantly inhibited by only one injection of ^131^I-EM@ALA. By comparing the toxicity of the ^131^I-EM@ALA and ^131^I-EM@PpIX groups, we confirmed that our strategy of using ALA, which is a photosensitizer precursor, effectively reduced the side effects of treatment. Our study provides a novel multi-therapeutic concept for antitumor treatment, as well as offering a new vision for synergistic radiotherapy and CR-PDT without an external light source.

## Supplementary Information

**Additional file 1: Figure S1.** Full radiochemical purity data of ^131^I-EM@ALA in PBS (**a**) and FBS (**b**). **Figure S2.** WB protein identification photograph of 4T1 lysate, EM, and EM@ALA. **Figure S3.** SPECT/CT imaging (left) and distribution (right) study were performed at 24 h after injection of ^131^I-EM@ALA injection. **Figure S4.** Cerenkov radiation imaging with different concentration of ^131^I without PpIX. **Figure S5.** Cerenkov radiation imaging in different kinds of solutions with different filters. **Figure S6. **Effect of ^131^I-EM@ALA treatment on apoptotic-related proteins in liver (**a**), and autophagy-related proteins (P62 and LC3) in 4T1 cell (**b**) and 4T1 tumor tissue (**c**) determined by western blot.

## Abbreviations

PDT: Photodynamic therapy
CR: Cerenkov radiation
CR-PDT: The Cerenkov radiation-induced PDT
ALA: 5-Aminolevulinic acid
ROS: Reactive oxygen species
EM: Exosome-mimetic
PpIX: Protoporphyrin IX
PSs: Photosensitizers
EPR: The enhanced permeability and retention
RT: Radiation therapy
FBS: Fetal bovine serum
TEM: Transmission electron microscope
WB: Western blot
iTLC-SG: The instant thin-layer chromatography-silica gel
SPECT/CT: Single-photon emission computed tomography/computed tomography
AST: Aspartate aminotransferase
ALT: Alanine aminotransferase
BUN: Blood urea nitrogen
CRE: Creatinine
ALP: Alkaline phosphatase
WBC: White blood cells
Neu%: Neutrophil percentage
Lymph%: Lymphocytes percentage
Mon%: Monocyte percentage
RBC: Red blood cells
HCT: Hematocrit
HGB: Hemoglobin
MCH: Mean corpuscular hemoglobin
MCV: Mean corpuscular volume
MCHC: Mean corpuscular hemoglobin concentration
PLT: Platelets
MPV: Mean platelet volume
RDW: Red cell volume distribution width
H&E staining: Hematoxylin and eosin staining
TUNEL staining: Terminal deoxynucleotidyltransferase-mediated dUTP-biotin nick end-labeling staining
SD: Standard deviation
GAPDH: Glyceraldehyde 3‑phosphate dehydrogenase
