# Supplementary material for: Minimizing adverse effects of Cerenkov radiation induced photodynamic therapy with transformable photosensitizer-loaded nanovesicles
Source: J Nanobiotechnology. 2022 Apr 27;20:203. doi: 10.1186/s12951-022-01401-0 (PMC9044600; doi:10.1186/s12951-022-01401-0)
Supplement: Supplementary file 1 — Additional file 1: Figure S1. Full radiochemical purity data of 131I-EM@ALA in PBS (a) and FBS (b). Figure S2. WB protein identification photograph of 4T1 lysate, EM, and EM@ALA. Figure S3. SPECT/CT imaging (left) and distribution (right) study were performed at 24 h after injection of 131I-EM@ALA injection. Figure S4. Cerenkov radiation imaging with different concentration of 131I without PpIX. Figure S5. Cerenkov radiation imaging in different kinds of solutions with different filters. Figure S6. Effect of 131I-EM@ALA treatment on apoptotic-related proteins in liver (a), and autophagy-related proteins (P62 and LC3) in 4T1 cell (b) and 4T1 tumor tissue (c) determined by western blot. [file 12951_2022_1401_MOESM1_ESM.docx]

***Supporting***

**Method of measuring radiolabeling stability in PBS and FBS of ^131^I-EM@ALA**

After the preparation of ^131^I-EM@ALA, we added the PBS or fetal bovine serum (FBS) to ^131^I-EM@ALA and put them in room temperature for 48h. During the 48h, the labeling rate of ^131^I were measured by thin layer chromatography (TLC) in 4h, 8h, 12h, 24h, and 48h. The instant thin-layer chromatography-silica gel (iTLC-SG) was used as the medium and normal saline as the developing agent. The detailed method of thin layer chromatography (TLC) is as followed.

1. Add 10 μl ^131^I-EM@ALA to the start point of the iTLC strip (at the first centimeter of the iTLC strip), and then dry the strip.

2. Then add 0.5 ml normal saline to a 10ml EP tubes and put the iTLC-SG into the tubes and allow the solvent to run up the iTLC strip.

3. When the solvent up to 2 cm from the top of the strip, remove it from the tubes and dry the strip.

4. Then lay the strip onto the radio-iTLC scanner in the appropriate orientation for the scan.

5. Finally, scan the radio-iTLC and calculate the amount of free ^131^I and ^131^I-EM@ALA.


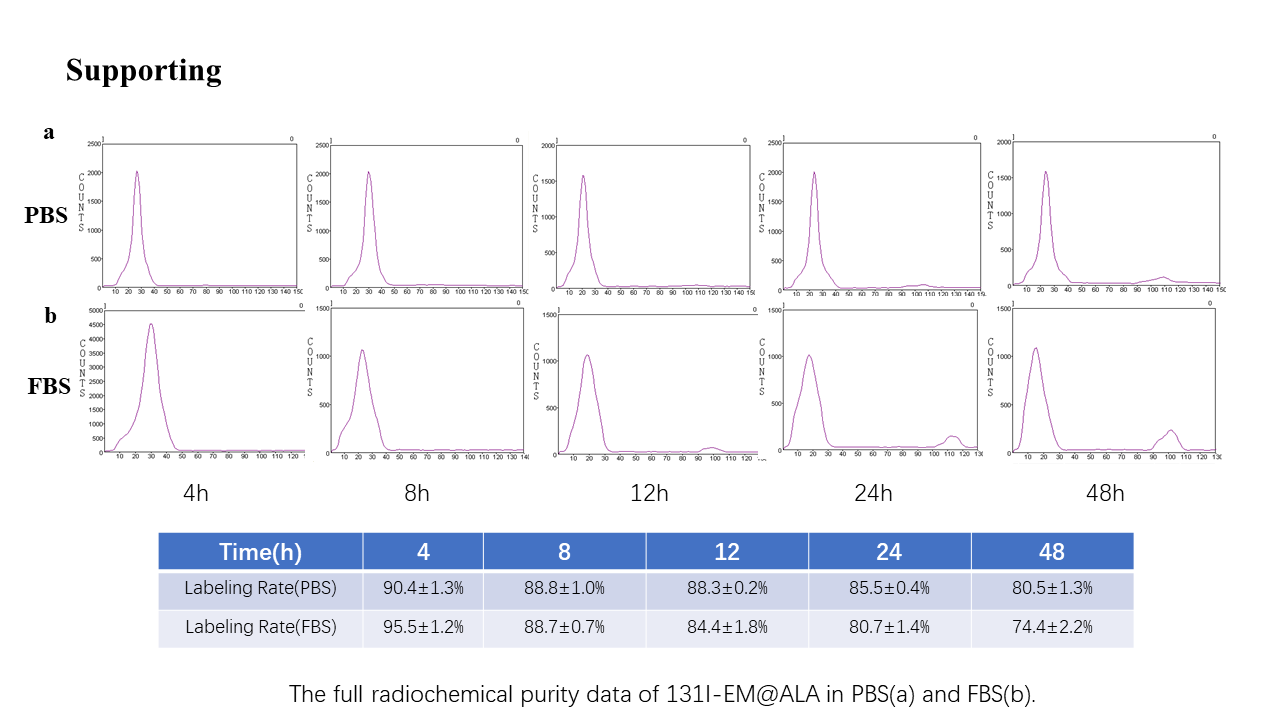


**Fig. s1** Full radiochemical purity data of ^131^I-EM@ALA in PBS (**a**) and FBS (**b**).


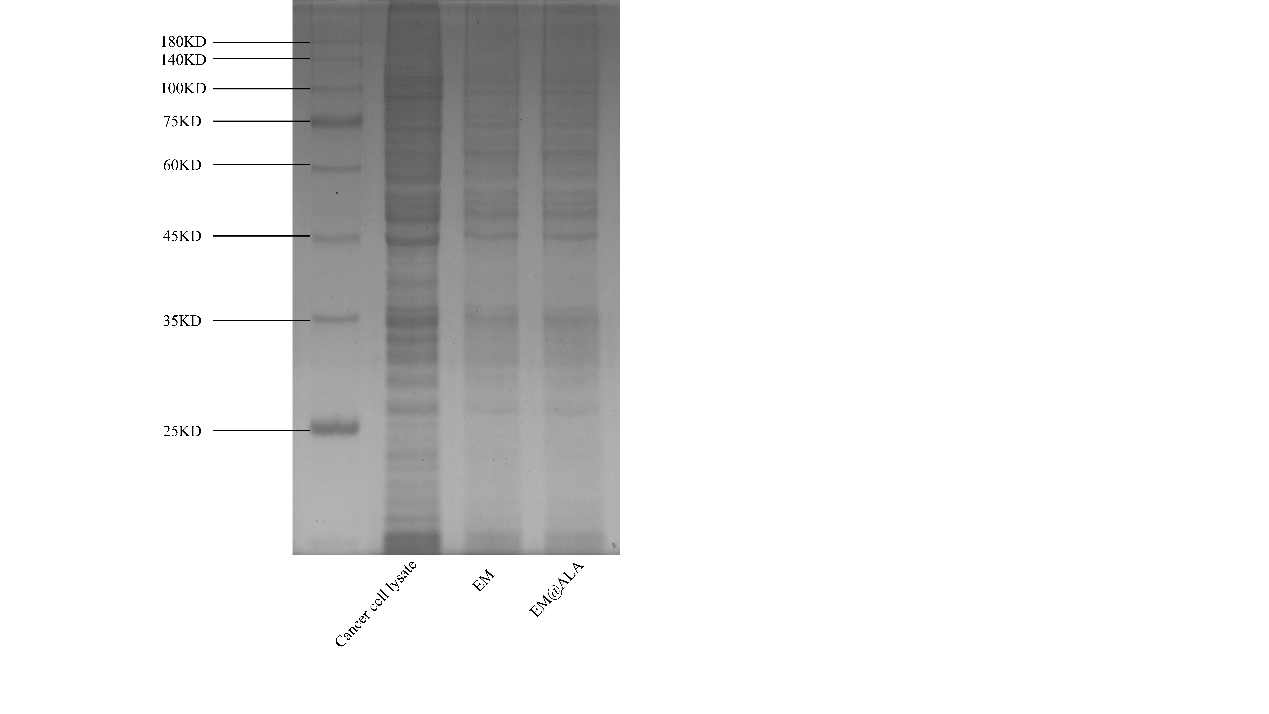


**Fig. s2** WB protein identification photograph of 4T1 lysate, EM, and EM@ALA.


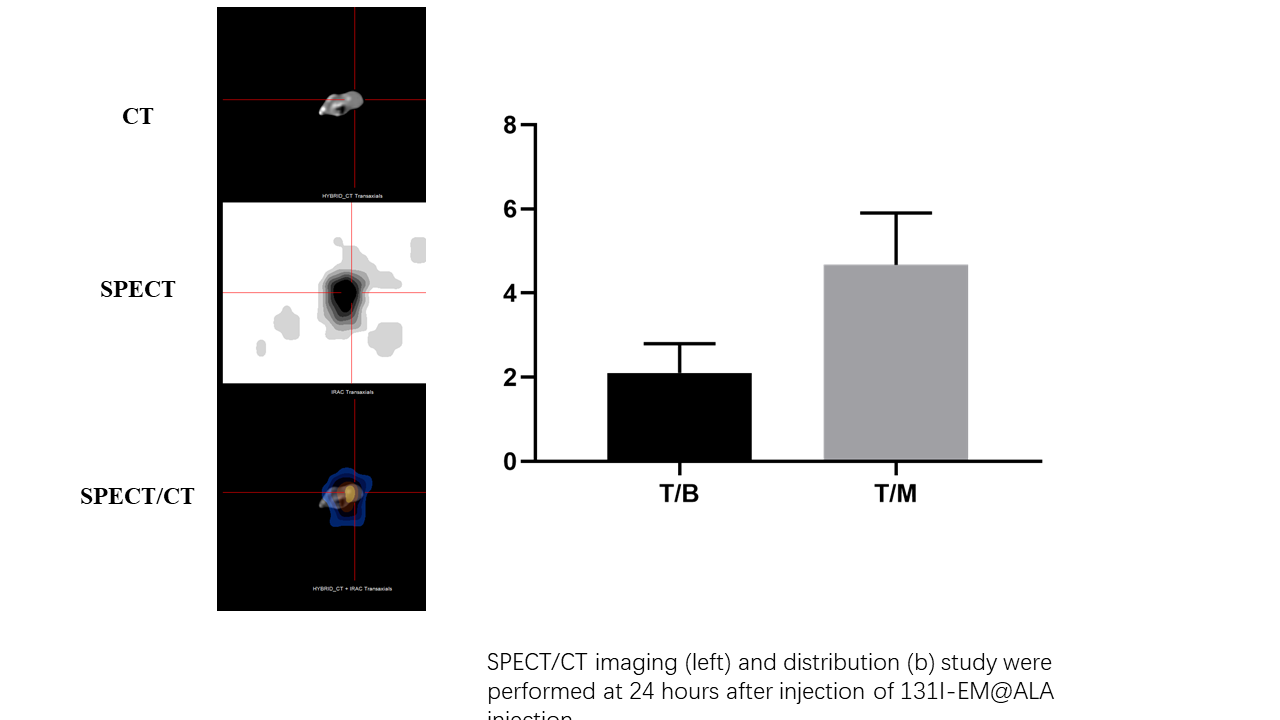


**Fig. s3** SPECT/CT imaging (**left**) and distribution (**right**) study were performed at 24 hours after injection of ^131^I-EM@ALA injection.


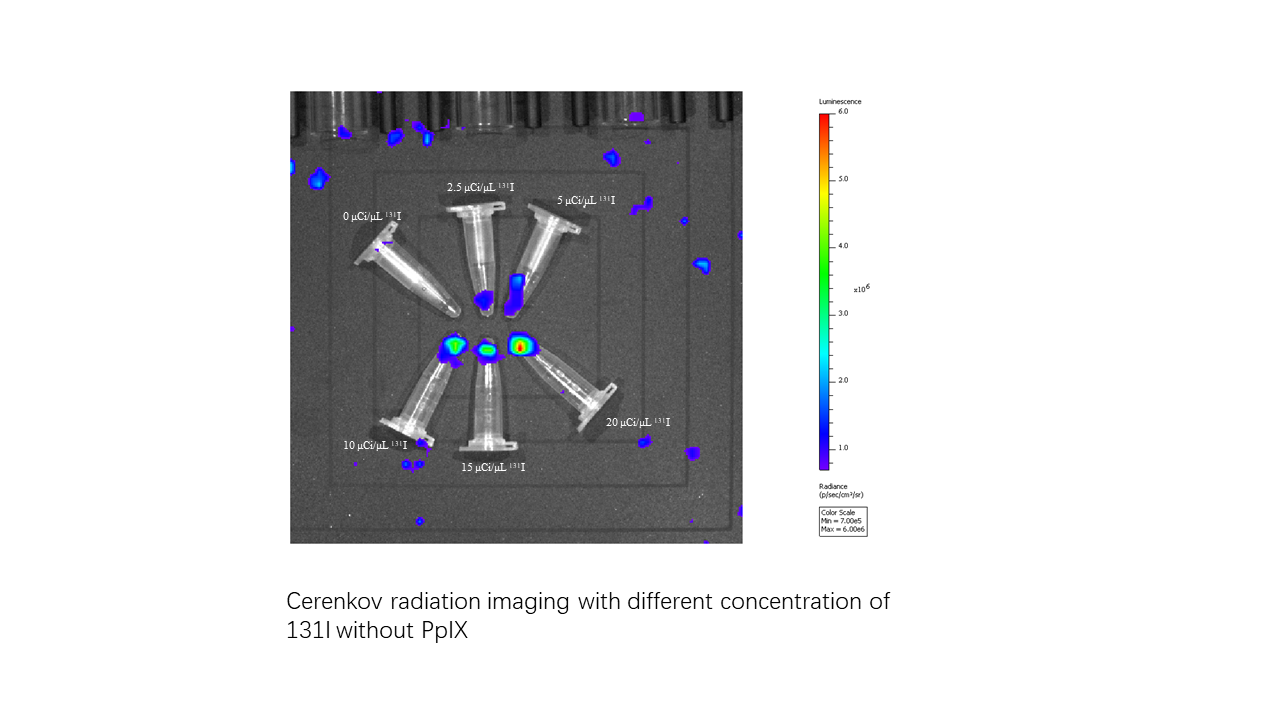


**Fig. s4** Cerenkov radiation imaging with different concentration of ^131^I without PpIX


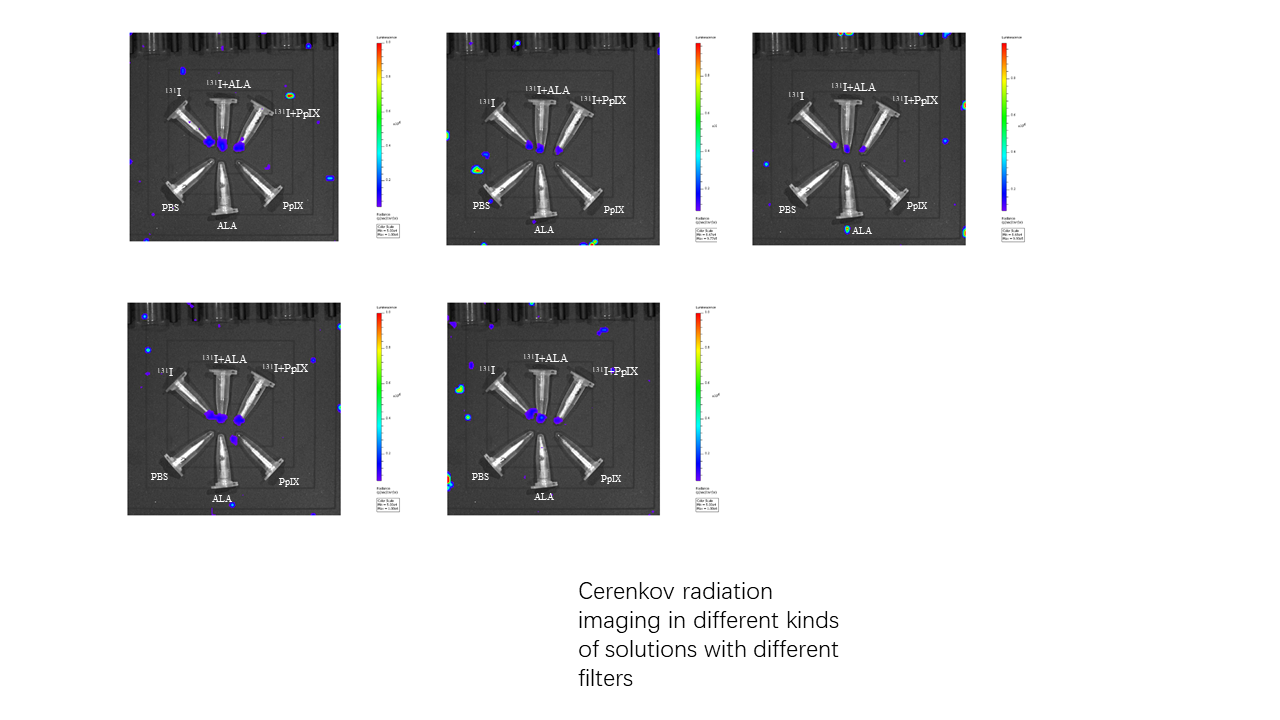


**Fig. s5** Cerenkov radiation imaging in different kinds of solutions with different filters


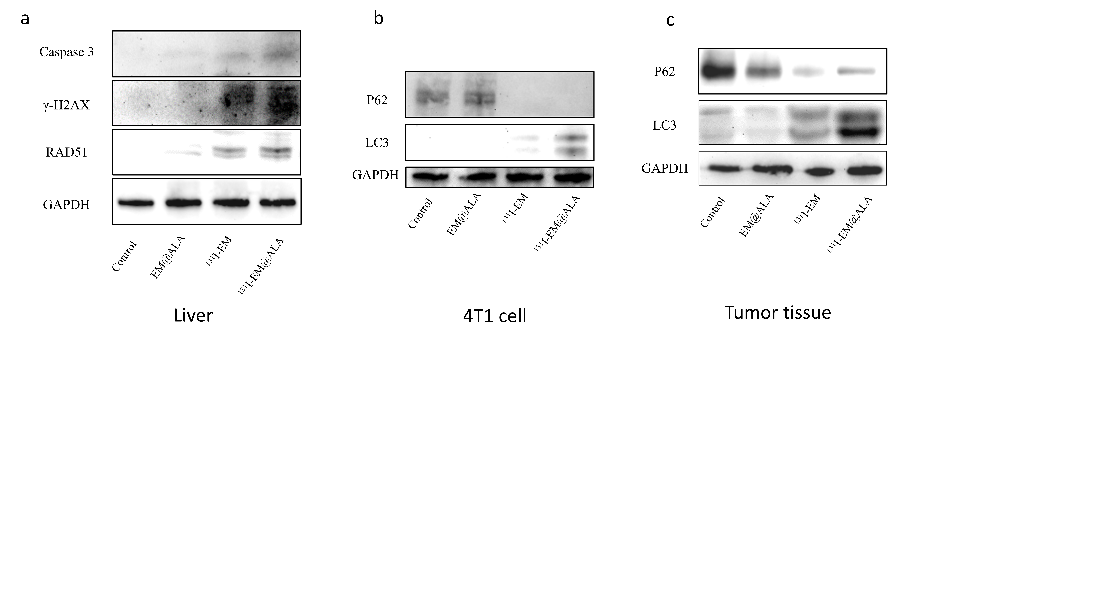


**Fig. s6** Effect of ^131^I-EM@ALA treatment on apoptotic-related proteins in liver (a), and autophagy-related proteins (P62 and LC3) in 4T1 cell (b) and 4T1 tumor tissue (c) determined by western blot
